# Impacts of Bovine Trace Mineral Supplementation on Maternal and Offspring Production and Health

**DOI:** 10.3390/ani10122404

**Published:** 2020-12-16

**Authors:** Megan Van Emon, Carla Sanford, Sarah McCoski

**Affiliations:** Department of Animal and Range Sciences, Montana State University, Bozeman, MT 59301, USA; carla.sanford@montana.edu (C.S.); sarah.mccoski@montana.edu (S.M.)

**Keywords:** cattle, colostrum, health, production, reproduction, trace minerals

## Abstract

**Simple Summary:**

The objective of this review is to address the importance of trace minerals in maternal and offspring health and production. Several specific trace minerals are discussed in the review, but many trace minerals are lacking research in the area of health, more specifically, maternal trace mineral impacts on offspring health. Much of the research focuses on production and growth and there is limited information regarding the impacts of maternal trace mineral supplementation and offspring health. Selenium, copper, zinc, and manganese have been researched more readily than others, such as cobalt and iron, which have had minimal research conducted.

**Abstract:**

Nutritional status can have major implications for animal health and production. Energy balance is easily determined using a body condition scoring system. This allows producers to readily adjust diets to meet an animal’s needs. Far less obvious is an animal’s trace mineral status, which is typically not assessed until an animal’s performance falls below expectation or illness is detected. Trace mineral toxicities and deficiencies can manifest as reduced thriftiness and/or poor reproductive performance, resulting in economic consequences for producers. Maternal mineral status not only impacts dam heath, but also the health of subsequent offspring. Both the oocyte and embryo are susceptible to changes in maternal mineral status. This susceptibility is maintained throughout fetal development via placental control of nutrient transfer to the fetal system. Furthermore, maternal mineral status continues to impact offspring health via colostrum and milk quality. Herein we discuss the roles of trace minerals in bovine reproductive performance, maternal health, colostrum and milk quality, and offspring health.

## 1. Introduction

Maternal health is highly dependent on trace mineral status of the animal. Trace mineral status changes throughout the year depending on several factors, including lactation, health, growth, feed quality, gestation progression, and pregnancy status. Minerals being transported for fetal development and subsequently neonate calf health rely on maternal mineral intake and status. Minerals are often offered free-choice in a loose or block form, but intake with these supplements is not highly regulated and can be highly variable. Several factors impact mineral intake, such as animal requirements, soil characteristics, forage type and availability, water quality, protein and energy supplements, mineral supplement palatability, source and physical form of the mineral supplement, freshness of the mineral, and mineral access [1]. Tait and Fisher [1] also noted that some animals did not consume mineral when offered free-choice. Because of the highly variable intake by cows on pasture, fetal and newborn calf mineral status may be altered, ultimately impacting fetal growth and development as well as neonate immune status.

Literature on trace mineral supplementation highlights the importance of evaluating an animal’s mineral status prior to supplementation, as reduced reproductive performance was reported in animals receiving copper (Cu), cobalt (Co), manganese (Mn), and zinc (Zn) at higher concentrations than NRC requirements prior to breeding [2]. Stokes and colleagues [3] found that repeated treatment with injectable trace minerals prior to artificial insemination (AI) had no effect on pregnancy rate in beef heifers. Additionally, a study in dairy cattle found that animals subjected to a two-dose trace mineral protocol, one prior to calving and a second prior to AI, had lower conception rates at first service [4]. Collectively, these studies suggest that supplementation above an animal’s requirements may have no benefit or may even be detrimental to reproductive performance.

Trace mineral supplementation has been widely studied; however, many of these studies studied several trace minerals or overall mineral nutrition. Additionally, many of the studies focused on supplementation post-weaning or during the feedlot phase. Little research has been conducted on maternal trace mineral supplementation and offspring health. Therefore, previous researchers have been summarized in this review with the main objective to provide information on the impacts of maternal trace mineral on reproduction and offspring health and production. 

## 2. Maternal Production and Health

### 2.1. Overall Mineral Nutrition

#### 2.1.1. Reproduction

Adequate reproductive efficiency is critical to the success of a livestock production operation but is the last priority for nutrient partitioning. It has been proposed that nutrient priority first addresses that of basal metabolism and reproduction function secondly [5]. There are inconsistent reports on the impacts of mineral supplementation on reproductive performance. Mineral supplementation of Cu, Zn, and Mn resulted in acceptable AI pregnancy rates yet variation from year to year was observed [6]. Supplementation with complex trace minerals (Zn, Mn, Cu, and Co) from day −30 to +30 of parturition increased endometrial expression of genes associated with inflammation, prostaglandin synthesis, and antioxidant response [7]. These findings may indicate faster uterine involution following parturition when trace minerals are supplemented during the pariparturition period in cattle.

There is little evidence that injectable trace minerals impact folliculogenesis. Treatment with an injectable trace mineral during the periconception period had no effect on antral follicle count or ovarian size in beef heifers [3]. Similarly, dietary trace mineral supplementation had no effect on the number of ova/embryos recovered from beef heifers, or the number of corpus lutea on the ovaries, indicating trace mineral supplementation likely does not impact ovulation [8]. While follicular activity may not be impacted by trace minerals, the oocyte appears to be susceptible to maternal dietary supplementation, as supplementation prior to ovum pick-up (OPU) increased the number of cumulous-oocyte complexes (COC) collected and increased oocyte maturation rates during in vitro culture [9]. 

Though follicular cells do not appear to be affected by trace mineral supplementation, luteal cell development and functions seem to be impacted. Injectable trace mineral administered 11 d prior to AI tended to decrease corpus luteum (CL) diameter, and decrease CL volume, though progesterone concentration was unaffected [10]. This suggests the cells of the CL may produce progesterone more efficiently following trace mineral supplementation. Additionally, animals receiving trace minerals had an increase in antioxidant enzymes. This may have reduced the amount of reactive oxygen species, which control steroidogenesis and progesterone release from the CL [11]. Collectively, these data suggest supplementation with injectable trace minerals improves reproductive performance by enhancing oocyte quality and CL function.

#### 2.1.2. Health

Overall mineral nutrition is crucial to maintaining health status in livestock. As would be expected, cows supplemented with organic or inorganic sources of Co, Cu, Mn, and Zn had increased liver concentrations of Co, Cu, and Zn compared with non-supplemented cows [12]. Although liver trace mineral concentrations increased, health impacts were not determined. Therefore, health status of supplemented animals could not be assessed. Cow body weight and body condition score were not impacted by inorganic or organic trace minerals supplemented at a low or high level; however, cows fed a high level of inorganic trace mineral lost more weight during the calving period than cows fed a high level of organic trace mineral [13]. This information suggests that organic trace minerals have a much larger impact on cows at calving than inorganic trace minerals. Maintaining body weight at calving can have an impact on colostrum quality and subsequently the health of the calf. This suggests that not only meeting trace minerals requirements is important to maintaining cow production, but the source of minerals as well. Having adequate trace mineral status aids in daily maintenance and has the potential to improve production and health. Supplementing Mn, Cu, and Zn during the 21-d prior to calving increased dry matter digestibility in dairy cattle compared to non-supplemented control cows [14]. Supplementing an organic methionine chelated source of Mn, Zn, and Cu increased total antioxidant capacity in cows immediately postpartum compared to cows supplemented with inorganic or glycine chelated or not supplemented with Cu, Mn, and Zn [14]. Additionally, immunoglobulin A and M concentrations were increased in cows supplemented with organic Cu, Mn, and Zn immediately post-calving compared to cows not supplemented [14]. These data suggest that cows supplemented with organic, chelated forms of trace minerals may have improved immune status and improved production.

### 2.2. Cobalt

#### 2.2.1. Reproduction

There has been little research conducted on the impacts of Co supplementation on cattle health and production. Currently, the mechanism by which Co acts to improve reproductive success is unclear, though current data indicate Co may impact ovarian behavior and embryogenesis. Work in sheep found that ewes supplemented with Co prior to embryo collection had a heightened response to the superovulation protocol [15]. Supplemented ewes also had a higher embryo quality grade compared to untreated ewes at the time of collection [15]. This suggests that Co plays a role in embryo development and embryo quality in sheep. More research needs to be conducted to identify the specific role of Co in bovine reproduction. 

Ruminant microbes have the ability to synthesize vitamin B12 from Co. While ruminal synthesis of B12 is sufficient to meet the needs of cattle, it is not adequate to prevent fluctuations in serum B12 concentrations observed around the time of parturition [15]. These fluctuations may impact subsequent reproductive performance, driving researchers to investigate the role of B12 supplementation on bovine reproduction. Duplessis et al. [16] found that multiparous cows supplemented with B12 in conjunction with folic acid had a 50% reduction in dystocia compared to untreated cows. Additionally, day of first breeding postpartum was 3.8 d earlier in supplemented cows compared to their untreated counterparts. Authors suggest this may be due to the vitamin supplementation improving energy balance, citing previous work which found that folic acid- and B12-supplemented cows experienced reduced BW and BCS losses following parturition [17]. These cows also had lower milk fat and higher milk protein, collectively suggesting an improved energy balance in supplemented animals. It is well understood that negative energy balance impairs several facets of reproductive performance [18], thus it is likely that this is the mechanism by which vitamin B12 acts to improve bovine reproduction. Of course, it is important to note that these studies examined the effects of B12 in conjunction with folic acid. The effects of B12 alone on reproductive performance remain to be investigated. 

#### 2.2.2. Health

One of the main functions of Co is as a component of vitamin B12, which is formed by rumen microorganisms. Because of this main function, Co is a required trace mineral, and may impact overall cow production and health. Vitamin B12 (cobalamin) functions through coenzymes, one-carbon metabolism, and gluconeogenesis [19]. These functions suggest that vitamin B12 is involved in energy metabolism and is important for rumen microbial synthesis of methane, methionine, and acetate [19]. Therefore, Co has an indirect role in these functions as well. Without synthesis of vitamin B12 within the rumen, energy and one-carbon metabolism are not efficient and can have detrimental impacts on beef cattle health and production.

Research on Co has had varying results when Co is supplemented above requirements. However, previous work has indicated that Co requirements may be too low [20,21]. Stangl et al. [21] suggested that Co requirements should be increased to 0.24 or 0.26 mg/kg of DM to maximize liver and plasma vitamin B12 concentrations, respectively. Additionally, Schwarz et al. [20] indicated that to maximize the body weight gain and feed intake, Co requirements should be increased to 0.12 or between 0.16 and 0.18 mg/kg of DM, respectively. However, it is difficult to determine if Co requirements should be altered based on these two recommendations. Stangl et al. [21] and Schwarz et al. [20] utilized growing bulls to establish the increased Co requirements. This does lend to the fact that there are currently no established trace mineral requirements for bulls. Moreover, it has been suggested that if Co needs are met, vitamin B12 synthesis within the rumen will be adequate.

### 2.3. Copper

#### 2.3.1. Reproduction

Studies utilizing Cu supplementation protocols highlight the necessity of Cu for pregnancy success as it is associated with normal reproductive function. Nazari and colleagues [22] found that dairy cattle exhibiting normal luteal activity had elevated concentrations of Cu compared to cows experiencing a prolonged luteal phase, short luteal phase, delayed ovulation, and anovulation. This study also reported that pregnant cattle had higher circulating concentrations of Cu compared to non-pregnant animals [22]. Copper’s association with luteal function and pregnancy status indicate it may play a role in the interaction between the CL and endometrium around the time of luteolysis. During the estrous cycle, the non-gravid uterus will synthesize prostaglandins which results in luteolysis and the start of the next follicular wave. A direct relationship between Cu deficiency and reduced prostaglandin synthesis has been identified in rats [23], though this relationship has not been established in cattle. Future work should aim to identify this possible connection, as luteolysis is critical to reproductive function in cattle.

Copper also appears to play a role in female gamete viability, as several reports on its effects on oocyte and embryo development exist. In vitro work found Cu supplementation had no effect on bovine oocyte maturation [24], however, cumulus cell apoptosis was reduced in bovine cumulus-oocyte complexes exposed to Cu during the maturation stage of development [25]. Moreover, Picco and colleagues [26] reported that Cu supplementation during in vitro maturation (IVM) decreased cumulus cell DNA damage. Though these studies highlight the beneficial role of Cu on oocyte development, it is important to recognize that Cu can have cytotoxic and genotoxic behaviors. Cumulus cells exposed to high Cu concentrations (≥120 ug/dL) had lower mitochondrial activity, heightened incidence of apoptosis, and increased DNA damage compared to unexposed cells [27]. Cumulus cells are critical to oocyte viability, as they provide nutritive support and protect against damage during maturation [28,29]. Therefore, Cu is required for oocyte viability, but there is also a maximum critical limit for improving oocyte quality. Copper’s positive effects on oocyte development are maintained through early embryogenesis. Supplementation of Cu during oocyte IVM increased embryo cleavage rates, the number of embryos reaching the blastocyst stage, total blastocyst cell number, and decreased the number of apoptotic blastomeres in the bovine blastocyst [24,26]. These findings are evidence of Cu’s role in oocyte and resulting embryo viability. Collectively, Cu may act by affecting multiple reproductive processes (luteolysis and embryogenesis) and on several cell types (endometrial cells, luteal cells, cumulus cells) to improve pregnancy retention in cattle. 

Copper deficiency is common in cattle, and breed differences in Cu metabolism have been reported. When fed Cu-deficient diets, Simmental cattle had Cu indices less than that of Angus [30,31]. Fry and colleagues [32] reported an impact of both diet and breed influencing Cu within the placentome, with cows fed a Cu-deficient diet and Simmental × Angus cows having reduced Cu concentrations in the placentome compared with cows fed a Cu-adequate diet and Angus cows, respectively. Breed and dietary Cu status affected placentome and fetal liver with Cu indices difference in Simmental cattle is attributed to lesser absorption when compared to Angus [30]. Copper requirements for the developing fetus increase, particularly during the last trimester with exponential growth of the conceptus occurring during this time as Cu is essential for proper fetal growth and development. Maternal Cu modulation does not compensate for poor Cu availability as findings have reported that both placenta Cu and fetal Cu concentrations were decreased in Cu-deficient diet fed dams [32]. This is particularly interesting as the placenta has a main role of maintaining fetal stage-specific mineral metabolism with an evident fetal-maternal gradient observed in the plasma levels of minerals. 

#### 2.3.2. Health

Copper deficiency has had mixed impacts on humoral or cell-mediated immunity. This may be due in part that Cu deficiency is primarily caused by antagonists such as Mo, S, and Fe. Additionally, because of the numerous interactions between Cu and several trace minerals, this makes elucidating Cu impacts on immune function difficult. However, it is important to note that pregnant cows have greater absorption and retention rates of Cu and Zn than non-pregnant cows [33]. However, Zn may impact Cu absorption, as both are absorbed through similar pathways, indicating a potential for competition between Zn and Cu for absorption sites. Cattle fed diets deficient in both Se and Cu or adequate Cu and deficient Se had a reduced number of neutrophils with the ability to kill Candida albicans compared to cattle consuming diets with adequate Se and Cu or adequate Se and deficient Cu [34]. Selenium (Se) and Cu interact to improve neutrophil activity and numbers to aid the immune system fight infection. However, Cu cannot overcome a Se deficiency, but Se may overcome a Cu deficiency in fighting infection. Additionally, Cu supplementation during gestation did not alter cow erythrocyte superoxide dismutase (SOD) activity [35]. Therefore, Cu may not play a critical role in the antioxidant activity but may have a role in overall immune system status. Additionally, Cu may play a larger role in immune system status in the presence of known antagonists compared with supplementation of Cu to potentially improve immune status.

### 2.4. Iron

#### 2.4.1. Reproduction

Iron (Fe) is an essential trace mineral for hemoglobin formation and thyroid hormone formation, and toxicity or deficiency symptoms are rarely observed in cattle. Iron also plays a role in cattle reproduction, and bovine gamete responsiveness to iron appears to be stage specific. Gao and colleagues [24] reported that Fe supplementation had no effect on bovine oocyte maturation in vitro. However, iron supplementation to embryo culture medium increased development rates to the 8-cell, morula, and blastocyst stages [24]. Furthermore, Fe supplementation reduced the number of apoptotic cells in the embryo [24]. These findings indicate that Fe has a role in both embryogenesis and embryo quality, though it may be nonessential earlier in development during oocyte maturation. Typically, Fe is not supplemented to livestock, as many of the feedstuffs consumed provide an adequate Fe diet. Care must be taken if excessive Fe is consumed as this may cause antagonistic effects with other trace minerals, such as Cu, Mn, Se, and Zn.

#### 2.4.2. Health

Iron serves as a main component of hemoglobin, where hemoglobin contains four Fe atoms, one in each of the four porphyrin rings [19]. Hemoglobin serves as the primary transporter of oxygen to the tissues within the blood. Therefore, if Fe would be deficient, oxygen transport would not be efficient or sufficient and may cause detrimental effects on the tissues. Iron also participates in several other processes, including immunity, energy metabolism, and blood production [36]. However, due to the abundance of Fe in many feedstuffs commonly fed to livestock and water, Fe supplementation is typically not required. Iron is readily retained in the body unless blood loss occurs. Additionally, Fe also plays a role in the inflammatory response in livestock and horses [37,38]. Moreover, attacking microbes tend to have a high affinity for Fe, and this elicits a response for haptoglobin to bind hemoglobin and reduces Fe availability to the attacking microbial population [39]. As stated previously, excessive Fe may cause detrimental impacts on other trace minerals, which may lead to negative impacts on livestock health and production.

### 2.5. Manganese

#### 2.5.1. Reproduction

Manganese has several functions throughout cattle production, including during gestation. Manganese deficiency is linked to silent heat, reduced conception, abortions, reduced birth weight, and an increased percentage of male calves [40]. Wilson et al. [41] reported that Mn supplementation increased pregnancy rates in cattle. One possibility is that its role in cholesterol production may regulate steroidogenesis during gestation [42]. Cholesterol is required for the synthesis of steroid hormones such as estrogen and progesterone, both critical to the establishment and maintenance of pregnancy. Additionally, the ovine CL contains manganese superoxide dismutase (SOD2) throughout its lifespan, indicating Mn may have a role in luteal progesterone synthesis in particular [43]. Luteal production of progesterone is critical to pregnancy success as it stimulates endometrial function to promote histotroph secretions, embryonic growth and conceptus elongation, and placentation [44]. Thus, it is possible Mn promotes pregnancy success via CL steroidogenesis by stimulating progesterone production by the corpus luteum, though further investigation into the mechanism is required.

There are also reports that Mn can act directly on the bovine cumulus-oocyte complex to enhance the oocyte and resulting in embryo viability. Anchordoquy and colleagues [45,46] report that cumulus cell DNA damage and apoptosis were decreased by Mn supplementation during oocyte maturation, though cumulus cell expansion was not affected. These positive impacts appear to be maintained through subsequent embryogenesis, as Mn supplementation during bovine oocyte IVM increased the blastocyst development rate. These embryos also had increased total cell number compared to untreated embryos, which is a common measurement of embryo viability [45]. While information on Mn activity post-fertilization is limited, available research indicates that the improvements in oocyte quality are sustained through the first several days of embryogenesis. 

Previous research has indicated that calcium and phosphorus may play a pivotal role in Mn status. Diets containing monocalcium phosphate reduced circulating Mn concentrations in growing dairy calves compared with diets that did not contain monocalcium phosphate [47]. Although this research was conducted in growing animals, this potential reduction in Mn may have a detrimental impact on reproduction, such as reduced conception rates and fertility. This reduction in conception rates occurred as the concentration of phosphorus increases and calcium is maintained [48]. Therefore, this research suggests that maintaining a proper calcium to phosphorus ratio is crucial to maintaining Mn utilization and absorption for reproduction. 

#### 2.5.2. Health

The primary role of manganese is reproduction and fetal development and may play a lesser role in health. Manganese concentrations may not be readily impacted in supplemented cattle. Marques et al. [12] observed similar liver Mn concentrations between cows supplemented with Co, Cu, Mn, and Zn and those that were not supplemented. Manganese retention has been shown to be dependent on the level of Mn excreted in the bile, which may suggest, based on the lack of Mn differences, that cows have efficient homeostatic control of Mn concentrations in the body [49]. Although Mn may not be significantly impacted by Mn supplementation, deficient Mn diets may lead to fetal deformities, such as twisted legs, shorter humeri, and enlarged joints [50,51]. Therefore, providing adequate Mn through supplementation is crucial for maintaining Mn concentrations within the body but over supplementation of Mn may not provide added health benefits.

An additional function of Mn is with superoxide dismutase, similar to Cu and Zn, which ultimately serves as an antioxidant [52]. Additionally, a multi-element injection that includes Mn has improved Mn superoxide dismutase activity in red blood cell lysate [53]. Moreover, based on the description by Suttle, reductions in Mn superoxide dismutase activity may be one of the first biochemical changes associated with Mn deficiency [19]. Previous research has also indicated that Mn plays a role in phagocytic activity in mice [54]. In addition, spleen cell antibody-dependent cell-mediated cytotoxicity was improved in mice receiving a single injection of Mn chloride compared with mice that received a single injection of saline [54]. These results suggest that Mn plays a critical role in cell-mediated immunity and maintaining adequate Mn concentrations aid in the health status of livestock.

### 2.6. Selenium

#### 2.6.1. Reproduction 

Selenium is a trace mineral that plays an important role in animal health and production, and Se deficiency is associated with reduced reproductive success [55]. Ovarian tissue, and follicles in particular, appear to be targets of Se. Ceko and others [56] utilized x-ray fluorescence imaging to determine that Se localizes to the granulosa cells of large bovine follicles, and its follicular expression is ten-fold higher than that in the CL. This specific localization of Se to large follicles and its known role in antioxidant defense suggest it may act by protecting the oocyte against oxidative stress and DNA damage during folliculogenesis. An additional mechanism for Se’s actions in bovine reproduction was emphasized by the in vitro work of Basini and Tamanini [57], who found that Se acts directly on granulosa cells to enhance their proliferation and increase estradiol synthesis. Estradiol production is critical to reproductive success because it acts on the hypothalamus to stimulate the release of gonadotropin-releasing hormone responsible for the preovulatory luteinizing hormone surge. Additional evidence of Se’s role in folliculogenesis was highlighted by work utilizing an undernourished ewe model. Grazul-Bilska et al. [58] demonstrated that Se deficiency resulted in reduced follicle cell proliferation, altered blood vessel and stromal tissue development of the fetal ovaries. Furthermore, in vitro studies have been successful in highlighting the importance of Se in oocyte and embryo development. Lizarraga and colleagues [59] reported that bovine oocytes matured in the presence of Se had a reduction in the number of apoptotic cells and increased embryo hatching rates compared to unsupplemented oocytes. Together, these studies indicate that Se plays a two-fold role in bovine reproduction, by acting on the follicle to promote folliculogenesis and by acting directly on the oocyte to protect against cell damage. Selenium likely has additional unidentified roles in reproduction, thus further research is needed. 

Selenium plays a role in the final stages of parturition, though the exact mechanism remains unclear. Early work by Brzezinska-Slebodzinska and colleagues [60] reported an association between Se-dependent glutathione peroxidase and the incidence of retained fetal membranes in dairy cattle. In this study, cows that experienced retained placentas for longer than 12 hours after parturition also had lower circulating concentrations of glutathione peroxidase up to two weeks prior to calving. These findings compliment the work by D’Aleo et al. [61] who reported a 20% incidence of retained placenta in cows fed a low Se diet. Additionally, previous work has demonstrated that Se concentrations in the cotyledon are increased in Se-supplemented cows [62]. This lends to the understanding that Se readily crosses the placental barrier during gestation and can potentially lead to increased Se concentrations in the calf.

#### 2.6.2. Health

Selenium is a main component of antioxidants, which protect against free radicals and lipid peroxidation. Selenium supplementation via sodium selenite or Se biofortified forage successfully increased liver Se and circulating Se concentrations compared with non-Se-supplemented control cows [62]. Additionally, grazing of Se biofortified forages for a 6-week period increased cow whole blood Se concentrations for 4- or 5-month post-grazing compared with non-Se-supplemented or sodium selenite-supplemented cows, respectively [63]. Similarly, Jaaf and others [64] observed an increase in blood and liver Se concentrations when supplemented with Se biofortified alfalfa. This research suggests that Se source may play a pivotal role in absorption and may influence circulating and storage concentrations.

Selenium is a major constituent of glutathione peroxidase, which aids in the reduction of hydrogen peroxide and other peroxide radicals. Moreover, Se is readily transferred to the fetus through the placental barrier at the expense of the cow [65,66]. During the third trimester, the fetus increases in weight and length exponentially, which can cause impacts on maternal Se status. A reduction in maternal Se status could lead to reduced antioxidant production and activity. Therefore, additional supplementation of Se during the third trimester may be needed to maintain maternal Se status and antioxidant activity. However, care must be taken with Se supplementation because the requirement for Se in beef cattle is quite low and there is a narrow range between toxicity and deficiency [67]. Cows supplemented with biofortified Se forage had an increased response to vaccination with J-5 Escherichia coli bacterin compared with cows supplemented with sodium selenite [63]. Cows supplemented with 45.5 mg of Se/d for 15 d pre-calving had increased red blood cell glutathione peroxidase activity compared to cows supplemented with 13.3 mg of Se/d [68]. Similarly, cows supplemented with Se via biofortified alfalfa had increased glutathione peroxidase concentrations compared to non-Se-supplemented cows [64]. Additionally, previous research has indicated that Se deficiency may play a role in severity and duration of mastitis, but also may be dependent on the bacterial species [69]. 

As has been previously discussed, Cu and Se work together and are crucial to maintaining neutrophil activity. Selenium has the ability to overcome or rescue neutrophil activity in cattle fed Cu-deficient diets [34]. However, this ability may be time sensitive in terms of length of time cattle are Cu deficient, as length of Cu deficiency increases, cadidacidal activity decreases even with supplemental Se [34]. Neutrophils are crucial to fighting infections and removing pathogens. Additionally, neutrophil glutathione peroxidase activity decreased in Se-deficient diets, even with adequate Cu, compared to cattle fed Se adequate diets with or without adequate Cu [70]. This was expected because of Cu being required for superoxide dismutase formation, which cannot be replaced by Se. However, SOD activity was not altered by Se or Cu being deficient or adequate, all cattle had a reduction in SOD activity during the 28-week study [70]. Selenium may partially overcome limited Cu to maintain SOD and glutathione peroxidase activity. Therefore, Se is crucial to maintaining and improving health status, and may overcome the impacts of other deficient trace minerals.

### 2.7. Zinc

#### 2.7.1. Reproduction

Zinc is well-recognized for its importance in reproductive performance. Nazari and colleagues [23] reported higher circulating zinc concentrations were associated with normal luteal activity and higher pregnancy rates. Conversely, cows experiencing an abnormal luteal phase, delayed ovulation, and pregnancy failure had reduced Zn concentrations [22]. The positive relationship between Zn concentration and pregnancy rate may be a result of Zn acting directly on the cumulus-oocyte-complex and embryo, thus creating a more viable pregnancy. In vitro work found Zn supplementation during oocyte culture reduced cumulus cell DNA damage and apoptosis compared to those cultured without Zn [71]. As previously discussed, cumulus cells are critical to oocyte vigor. It seems that exposure to Zn even prior to fertilization is necessary for pregnancy success. Furthermore, embryos exposed to Zn during in vitro oocyte maturation had increased cleavage rates and an increased percentage reaching the blastocyst stage, though these blastocysts had a reduction in total cell number compared to unexposed blastocysts [72]. More recent work found that the post-fertilization gamete is responsive to Zn as well. Zinc supplementation during the first 8 d post-fertilization resulted in an increase in inner cell mass (ICM) and total cell numbers, an indication of higher embryo quality [73]. These findings indicate that the benefits of Zn supplementation are dependent on the timing of exposure. 

Proper fetal growth requires sufficient maternal Zn intake at pre-breeding and through gestation as mice fed a Zn-deficient diet during pregnancy resulted in a 26% reduction in circulating Zn and an 8% reduction in near term fetal and placental weights [74]. Wilson and colleagues [74] reported a decrease in maternal mean arterial pressure during late gestation with implications of reduced blood flow and nutrient delivery to the placenta. Placental blood flow is necessary for transport of oxygen, water, and nutrients in late gestation [75]. It is proposed that negative impacts on the placenta and maternal cardiovascular system likely results in the *in utero* programming of offspring and development.

Zinc may also target the reproductive tract by improving the reproductive health in the postpartum period. Campbell and Miller found that days to first observed estrus was reduced in cows receiving Zn supplementation compared to non-supplemented animals [76]. Supplemental Zn also reduced the number of days to first AI but had not impact on days open. Authors suggest supplementation may act on the localized uterine immune response or by limiting uterine oxidative stress. This may subsequently hasten uterine involution and return to estrus. 

#### 2.7.2. Health

Zinc has many functions in the body, including protein synthesis, nucleic acid synthesis, carbohydrate metabolism, a major constituent of several metalloenzymes, and plays a role in several biochemical reactions. It has previously been suggested that immune function of ruminants may not be severely impacted by marginal Zn deficiency, but a severe deficiency may have detrimental impacts on cell-mediated and humoral immune functions [69]. Pregnant cows have greater apparent absorption and retention rates of Zn than non-pregnant cows [33]. Additionally, Hansard and others [77] observed that fetal concentrations of Zn increased 13 times between the first and second trimesters and 7 times during the third trimester. This previous research suggests that fetal Zn metabolism may be altered because of fetal growth demand. As cattle progress in gestation, Zn metabolism is altered in favor of fetal development. This has previously been observed by Hansard et al. [77], where fetal liver and bone Zn^65^ concentrations increased 3-fold at 24, 96, and 168 h post-intravenous dosing and maternal liver Zn^65^ concentrations decreased. The reduction in maternal liver Zn suggests that Zn is being mobilized for fetal development during gestation and strongly favors fetal liver storage. Additionally, cow body weight increased linearly at calving as the proportion of organic Zn was increased in the diet, but those cows also lost more weight during the first 4 weeks post-calving [78]. This suggests that Zn plays a role in maternal production and growth during late gestation and transfer of Zn to the fetus but may not impact calf birth weight. 

## 3. Colostrum and Milk Quality

### 3.1. Overall Mineral Nutrition

Colostrum quality and quantity has a significant impact on calf health immediately post-calving and prepares the immune system for growth and production. Colostrum quality is influenced by several factors, such as genetics, age, breed, and nutrition. However, overall nutrition may not have the greatest impact on quality, with cows grazing low-quality forage without supplementary feed, quantity was reduced, but quality was not significantly affected compared to cows receiving supplemental feed [79]. To ensure good colostrum quality, cows may have homeostatic mechanisms to maintain consistent quality for newborn calf health. Additionally, in dairy cows, mineral concentrations are greatest colostrum and milk immediately following parturition and decrease rapidly over 72 h, with the exception of potassium, which remains constant [80]. More specifically, it has previously been noted that mineral type (organic or inorganic) did not significantly alter trace mineral concentrations [81]. In contrast, a study conducted by Formigoni et al. [82] indicated that dairy cattle consuming organic trace minerals had increased immunoglobulins and tended to have increased Ca and Mg in the colostrum compared to cows consuming inorganic trace minerals. If minerals are greatest immediately following parturition, then it is critical that calves nurse within the first 6-hr post-calving to receive needed minerals. In lambs, when Zn or P were not supplemented, colostrum production was decreased through 18 h post-partum, but total immunoglobulin G (IgG) concentrations were not altered [83]. Therefore, mineral supplementation or lack thereof may alter the total colostrum yield but may not negatively impact the colostrum quality. Supplementing organic sources of Mn, Cu, and Zn increased colostrum quantity during the first two milkings but did not alter the composition [14].

### 3.2. Copper

Previous research has indicated that Cu potentially plays a larger role in circulating Cu concentrations through ceruloplasmin but may play a lesser role in immune status or colostrum/milk quality. However, there has been minimal research conducted with Cu supplementation impacts on colostrum or milk quality. Copper supplementation did not alter cow colostrum or milk Cu or Zn concentrations compared to non-supplemented cows [84]. Additionally, Cu supplementation only increased colostrum IgG concentrations in year 1, IgG concentrations were not different between maternal Cu treatments in year 2 [84]. Copper may only cause significant impacts to immune status or colostrum quality during times of Cu deficiency. When cows have adequate Cu status, additional Cu supplementation may not provide added benefits to colostrum and milk quality. 

### 3.3. Selenium

Selenium plays a major role in immune status of livestock, especially with its ability as an antioxidant. Cows supplemented with organic Se had greater colostral Se concentrations compared with cows supplemented with inorganic Se or not supplemented with Se [85]. Selenium secretion and synthesis is regulated in by mechanisms in the mammary gland, where Se concentrations are greatest in the colostrum and decrease as lactation progresses [86]. Contrary to previous research, these differences in Se concentrations in the colostrum also continued through week 6 and 12 post-calving [85]. Additionally, ewes drenched orally with Se had increased concentrations of Se in colostrum and milk compared to ewes not supplemented with Se [87]. Selenium-supplemented cows via biofortified alfalfa had increased colostral Se concentrations compared with non-Se-supplemented cows, but this rapidly decreased by 4-d post-calving and were similar to non-Se controls [64]. However, Awadeh et al. [88] observed no differences in Se colostrum or milk concentrations between Se-supplemented and non-supplement cows in the first 132 d of supplementation. Moreover, after continuing on the treatments through the next parity, Se concentrations were only increased in cows fed Se yeast compared with non-Se-supplemented controls [88]. This previous research may suggest that livestock have an efficient control mechanism for Se retention. This is further explained by the minimal daily dietary needs of Se in cattle [66]. 

This previous research may suggest that livestock have an efficient control mechanism for Se retention. This is further explained by the minimal daily dietary needs of Se in cattle [66]. Colostral IgG concentrations were increased in cows supplemented with 60 mg/kg sodium selenite and 60 mg/kg Se yeast compared with non-Se-supplemented controls [88]. Similarly, Ranches and others [62] did not observe differences in colostrum or milk Se or IgG concentrations. However, in other research, maternal supplementation of Se did not alter IgG or IgM concentrations in colostrum [66]. Additionally, milk glutathione peroxidase activity was not altered by maternal Se supplementation via biofortified alfalfa [64]. The mixed results on colostrum and milk quality observed when supplementing Se may indicate that source and Se status may have greater impacts than Se supplementation alone. 

### 3.4. Zinc

Previous research has indicated that Zn supplementation increases Zn concentrations in milk [89,90]. However, as supplemental Zn increases from 692 to 1279 mg/kg in the diet, no significant increases in milk Zn were observed [90]. Similarly, supplemental Zn did not alter milk Zn concentrations in dairy cattle [91]. Although Zn concentrations in milk were not altered, after three months of Zn supplementation, somatic cell counts were reduced compared to non-Zn-supplemented dairy cows [91]. Additionally, previous research has indicated that increasing the proportion of organic Zn in the diet of dairy cattle tended to increase IgG concentrations in colostrum [78]. There have been mixed results on the impact of Zn on colostrum or milk quality and may depend on maternal Zn status. Zinc has the potential for improving Zn concentrations in the colostrum and milk which may lead to improved IgG concentrations in the colostrum, ultimately impacting offspring health and production.

## 4. Offspring Health and Production

### 4.1. Overall Mineral Nutrition

Calves rely on placental transfer of nutrients during fetal development and colostrum and milk nutrient concentrations immediately post-calving. Additionally, colostrum serves as the main source of minerals for the newborn calf [92]. If colostrum mineral concentrations are altered or deficient immediately post-calving, calves may also have reduced mineral concentrations, which could negatively impact health and production. Previous research indicates that calf plasma mineral concentrations are altered more by calf age than by parity of the dam [80]. Although circulating trace mineral concentrations may not be significantly altered by maternal supplementation, calf liver stores of trace minerals may be positively impacted.

Calves born to cows supplemented with Co, Cu, Mn, and Zn had increased liver concentrations of Co, Cu, and Zn at birth compared to calves born to non-supplemented cows [12]. Calf birth weight was not impacted by maternal late gestation supplementation of Co, Cu, Mn, and Zn [12]. Although trace mineral status concentrations do not account for age of the calf/cow [93], calves from both Co-, Cu-, Mn-, and Zn-supplemented and non-supplemented cows were considered adequate in Cu and Zn. Manganese concentrations are difficult to alter in cattle and supplementation does not necessarily indicate Mn status. Previous research is indicative of this difficulty to alter Mn liver stores, as Mn concentrations were not different between calves born to Co-, Cu-, Mn-, and Zn-supplemented and non-supplemented cows [12]. 

Calf birth weight was not altered by late gestation trace mineral supplementation to cows, but weaning weight was increased in calves born to cows supplemented at a high level with organic trace minerals compared to calves born to cows supplemented at a low or high level with inorganic trace minerals [13]. Therefore, trace mineral source and bioavailability may play a role in improving calf growth from birth to weaning. Calves rely heavily on maternal milk production and quality from birth to weaning to consistently supply nutrients, including trace minerals. Maternal supplementation of trace minerals during gestation may influence post-calving growth through fetal programming and additionally through potential milk quality, which may subsequently lead to improved growth from birth to weaning. 

Source and bioavailability of trace minerals in a supplement program may also lead to improved immune status. Organic supplementation of Cu, Mn, and Zn for 60 d prior to calving increased calf serum total antioxidant capacity and immunoglobulin A and M concentrations compared to calves from non-supplemented cows [14]. Although health parameters were altered at 3 d of age by maternal mineral supplementation, circulating concentrations of Cu, Mn, and Zn were not altered by maternal Cu, Mn, and Zn supplementations [14]. Alternatively, neutrophil and lymphocyte concentrations were not altered in calves because of maternal trace mineral supplementation during late gestation [13]. Previous research on supplementing several trace minerals has led to mixed results on immune status. This could be due to the potential trace mineral interactions, such as the presence of antagonists, or maternal trace mineral status. Therefore, supplementation of individual trace minerals may provide specific benefits to offspring immune status.

### 4.2. Copper

Copper has several functions, including growth, reproduction, and health. Direct supplementation of Cu to livestock has been readily evaluated, but indirect supplementation, such as maternal supplementation for offspring development has been minimal. Maternal copper supplementation pre-calving did not significantly alter calf Cu concentrations at 10 d of age [84]. However, this comes as a surprise as maternal liver Cu concentrations in the non-supplemented group had reduced Cu concentrations [84] and would have been considered clinically deficient [93]. Therefore, this research suggests that cows have efficient mechanisms of shuttling needed Cu into the fetal liver to ensure adequate concentrations for health and production post-calving. Calves from Cu-deficient cows had marginal to sufficient Cu liver concentrations and were similar to calves from cows supplemented with Cu [84]. Maternal Cu supplementation did increase IgG concentrations in the calf at birth in a single year, but concentrations were not different in the second year [84]. Morbidity was not altered in calves because of maternal supplementation of Cu or lack thereof [84]. Moreover, maternal Cu supplementation, regardless of source, did not alter calf growth through weaning [84]. Therefore, Cu status of the dam and offspring may play a crucial role in the impacts of Cu supplementation on offspring health and development.

Antagonists play a large role in Cu status and potential health impacts. Leukocyte, white blood cells, concentrations were increased at 7 d of age in calves born to cows fed a Cu-deficient diet with additional Mo compared to calves born to cows fed a Cu-deficient diet with additional Fe or a Cu adequate diet [94]. However, by d 70 of age, calves born to cows fed a Cu-deficient diet without additional Fe, Mo, or Cu had increased leukocyte concentrations compared to calves born to cows fed a Cu-deficient diet with supplemental Fe or Cu [94]. Although concentrations of leukocytes were impacted by Cu deficiency and Cu antagonist supplementation, concentrations of neutrophils and lymphocytes were not altered [94]. This research suggests that antagonists of Cu may impact immune response in calves from Cu-deficient cows, by increasing leukocytes.

Copper concentrations may play a role in antioxidant activity, through SOD activity, which could lead to a role in immune function. Calf erythrocyte SOD activity was correlated (*r* = 0.42) with their respective dam erythrocyte SOD activity in the first week post-calving, but this correlation was reduced as calves aged [35]. Although dam and calf SOD activity were correlated, calf erythrocyte SOD activity was not impacted by cow Cu supplementation [35]. Previous research has indicated that dairy calves with acute diarrhea had increased circulating concentrations compared to healthy calves [95]. Acute diarrhea may have increased ceruloplasmin, which is an acute-phase protein that contains Cu, therefore, increasing circulating Cu concentrations [95]. Although previous research indicated that Cu did not impact SOD activity, ceruloplasmin was potentially impacted, which suggests that Cu may play a role in antioxidant protection against oxidative stress. 

It is interesting to note that correlation coefficients between cows and calves change throughout the supplementation period [13]. For example, correlations of liver Cu concentrations tended to be negatively correlated (*r* = −0.31) at calving and positively correlated (*r* = 0.38) 4 months later [13]. The negative correlation at calving may indicate that the cow is readily drawing on Cu liver storage to increase Cu content in the milk to increase the calf liver storage. The positive correlations observed 4 months post-calving may indicate that the dam is readily storing Cu in the liver, while maintaining an adequate supply in the milk for the calf. Additionally, the calf may be consuming forages and mineral supplements available at 4 months of age, which would allow the cow to adequately store Cu in the liver, and reduce the demand for trace minerals, specifically Cu, from the cow.

### 4.3. Selenium

Previous research has indicated that Se readily crosses the placental barrier into fetal tissues [65,66]. Lambs from ewes supplemented with Se had increased Se concentrations in whole blood, and skeletal muscle compared to lambs from ewes that were not supplemented with Se [87]. Similar results were observed by Taylor et al. [96], with fetal liver, muscle, plasma, and kidneys having increased concentrations of Se as Se supplementation increased in the ewe. Additionally, lambs born to ewes supplemented with Se had increased glutathione peroxidase activity [66]. Moreover, calves born to cows supplemented with Se had increased plasma Se concentrations; however, no differences were observed in liver or whole blood Se [62]. Since Se can readily cross the placental barrier, calves have the potential to have increased Se concentrations and the potential for improved health status compared to calves that are born to cows without adequate Se during gestation.

Although Se can readily cross the placental barrier, there have been mixed results on if this equates to increased antioxidant activity in the calves. Supplementing cows with 120 mg/kg sodium selenite or 60 mg/kg Se yeast increased IgM concentrations in calf serum, and cows consuming 120 mg/kg sodium selenite had calves with increased IgG concentrations compared with non-Se-supplemented controls [88]. One of the main functions of Se is the inclusion in selenoproteins, which play a large role in antioxidants. These antioxidants play a critical role in immune status in removing reactive oxygen species [19]. At 15 d of age, calves from cows supplemented for 15 d pre-calving with 45.5 mg of Se/d had increased glutathione peroxidase activity compared to calves from cows supplemented with 13.0 mg of Se/d [68]. In contrast, dairy calves born to cows supplemented with Se via biofortified alfalfa had similar glutathione peroxidase activity to calves born to non-Se-supplemented cows at birth and day 25 post-calving [64]. Additionally, calves born to cows supplemented with Se had similar concentrations of glutathione peroxidase activity and plasma IgG compared with calves born to non-Se-supplemented cows [62]. Because of these varying results, Se status may play a critical role in determining if glutathione peroxidase activity may be improved. Selenium and immune status may be further influenced by Se source. However, this potential increase in glutathione peroxidase activity from Se supplementation may lead to improved immune status of calves, which could reduce illness and potentially reduce treatment costs; therefore, leading to improved growth and production of those calves.

### 4.4. Zinc

Severe Zn deficiency in calves leads to slow healing wounds and can lead to secondary infections [97]. Additionally, Zn deficiency can cause joint issues, nose and mouth lesions, and cracking of hooves [97]. Although these symptoms may not directly impact animal health, the secondary infections that can occur do have an overall impact on health. Lambs born to ewes fed Zn-deficient diets for the final 115 d of pregnancy had reduced birth weights, liver weight, carcass Zn concentrations, liver Zn concentrations, and pancreatic Zn concentrations [98]. Although it has been demonstrated that Zn concentrations increase in the fetus during gestation [77], fetal body weight [77] or calf birth weight [78] has not been altered by Zn supplementation. Although overall health status was not evaluated, Zn concentrations were reduced, which could negatively impact immune system function or production. 

Previous research has indicated that dairy calves suffering from acute diarrhea had reduced circulating Zn concentrations compared to health calves [95]. The authors theorized that this may be due to the fact that calves suffering from diarrhea may have reduced Zn absorption and may subsequently cause a reduction in health status [95]. Additionally, erythrocyte lipid peroxide concentrations were also increased in calves with acute diarrhea, which may be indicative of the reduced Zn, which promotes antioxidant enzyme formation [95]. This suggests that Zn plays a role in antioxidant synthesis, which directly impacts health status of livestock. Therefore, during times of acute stress and illness, adequate Zn concentrations are needed to overcome excessive Zn excretion due, maintain antioxidant enzyme synthesis, ultimately reducing the harmful effects of oxidative stress, such as diarrhea.

## 5. Conclusions

Trace minerals greatly impact cattle health and performance. As reviewed above, maternal heath, reproductive performance, colostrum and milk quality, and offspring health are each susceptible to mineral supplementation either directly or indirectly (alterations in enzymatic function, DNA replication, antioxidant formation, etc.). However, several factors may impact the effectiveness of supplementation. First, it is essential that the mineral status of both dam and offspring be known prior to supplementation, as several minerals can have no added benefit or even be detrimental if supplemented in excess. Additionally, it is important to understand the targets and function of the discussed minerals. Mineral needs are not constant throughout an animal’s lifetime, but rather fluctuate depending on an animal’s physiological state (pregnancy status, lactation status, age, etc.). This impacts the timing of when specific minerals should be supplemented, whether it be during gestation, following parturition, or during early life. Using this information, producers can ensure they supplement minerals when they are both biologically and economically beneficial. 
